# Accommodations for Autistic Young Adults in Employment Processes: Experiences in Chile

**DOI:** 10.1192/j.eurpsy.2025.1806

**Published:** 2025-08-26

**Authors:** J. Madrid Lira, M. Ulloa Velascos, M. S. Burrone

**Affiliations:** 1 Instituto de Ciencias de la Salud; 2 Psychology Bachelor’s Degree, Escuela de Ciencias Sociales, Universidad de O’Higgins, Rancagua, Chile

## Abstract

**Introduction:**

Access to employment is a significant challenge in adulthood, particularly for autistic individuals. Accommodations, both in the workplace and during selection processes, are essential to support the inclusion of this population in the labor market. Given the dynamic nature of support needs, understanding the specific characteristics and needs of autistic individuals is necessary to provide reasonable adjustments. However, this process becomes more complex when individuals do not disclose their autism diagnosis, hindering organizations’ ability to identify and address their needs.

**Objectives:**

To identify accommodations that support the employment of autistic young adults in Chile.

**Methods:**

Semi-structured interviews were conducted with 12 autistic young adults with job interview experience in Chile. Participants, residing across five regions, were selected through theoretical sampling. Data were analyzed using content analysis in alignment with the principles of Grounded Theory by Glaser and Strauss. Ethical approval was obtained from the Universidad de O’Higgins Ethics Committee, and all participants provided informed consent.

**Results:**

While accommodations are deemed necessary by autistic individuals, they are seldom requested due to concerns that such requests may imply reduced competence, potentially lowering employment prospects. Participants in this study highlighted key dimensions essential for support, including procedural adjustments (advance preparation, privacy, and flexibility), interpersonal approaches (empathy and respect for diversity), and sensory accommodations (specific sensory considerations and clear, literal communication). Additionally, virtual interview formats were viewed positively, as they facilitate the fulfillment of certain sensory needs.

**Conclusions:**

This study reveals a tendency among autistic individuals not to request accommodations during job-seeking processes, despite recognizing their necessity. Promoting professional practices based on updated, inclusive knowledge is essential for developing employment processes that effectively support autistic individuals.

**Disclosure of Interest:**

None Declared

